# Physicochemical, Morphological, and Functional Properties of Starches Isolated from Avocado Seeds, a Potential Source for Resistant Starch

**DOI:** 10.3390/biom12081121

**Published:** 2022-08-15

**Authors:** Jiashui Wang, Yanxia Li, Zhiqiang Jin, Yunjiang Cheng

**Affiliations:** 1 Key Laboratory of Horticultural Plant Biology, Ministry of Education, Huazhong Agricultural University, Wuhan 430070, China; 2 Key Laboratory of Genetic Improvement of Bananas, Haikou Experimental Station, Chinese Academy of Tropical Agricultural Sciences, Haikou 570102, China; 3 National R&D Center for Citrus Preservation, Wuhan 430070, China

**Keywords:** avocado seed starch, morphology, physicochemical properties, in vitro digestibility, resistant starch

## Abstract

This study compared the physicochemical and functional properties of starches from eight cultivars of avocado seeds. Amylose content, morphology, crystalline structure, swelling power, solubility, thermal and pasting properties, and in vitro digestibility were investigated. The results revealed that the apparent amylose content of starches from avocado seeds varied with different varieties. Light microscopic and scanning electron microscopic examination demonstrated that the eight starches differed slightly in terms of morphology and granule size. The X-ray diffraction and Fourier transform infrared spectroscopy analyses showed that the crystal structure and chemical linkage of the avocado seed starches were similar. However, the pasting, water solubility, and thermal properties of the eight avocado seed starches differed. Importantly, all the starches had high resistant starch content (>60%), with the highest found in *Hass* seeds (77.83%). To conclude, starch from avocado seeds has a high potential for use in the production of resistant starch.

## 1. Introduction

Avocado (*Persea americana* Mill.) is a tropical and subtropical fruit native to Mexico and Central America. In recent years, avocado has gained popularity worldwide due to its multitudinous benefits to humans. Avocado is rich in unsaturated fats, proteins, minerals, vitamins, and bioactive compounds, and can provide a feeling of fullness. It is frequently used to make meal replacement products. Furthermore, avocado is a type of high-energy but low-sugar fruit, which well meets some demand for healthy foods [1]. Avocado also contains a variety of other beneficial compounds, including tocopherols [2], plant sterols, dietary fiber, and folic acid [1]. Unlike many fruits that have a sweet or acidic taste, avocado has a buttery consistency, a unique smooth and refreshing taste, and is widely consumed. As such, avocado has a wide range of applications in food, cosmetics, and pharmaceutical industries, including in the production of ice cream, mayonnaise, and sandwich spreads. However, processing avocado fruit produces a significant amount of waste, particularly the discarded seeds, which account for nearly 16% of the dry fruit weight [3]. These by-products can pose a negative impact on the environment, particularly the reproduction of pests. They also cause extra economic loss as a result of the expenditure of transporting them to disposal sites [4]. Therefore, the potential use of discarded avocado seeds not only adds value to fruit production in developing countries, but reduces environmental issues associated with seed handling.

Some researchers have looked into the starch extracted from avocado seeds. For example, Kahn [5] conducted the first research on avocado seed starch and investigated the morphology, gelatinization temperature, viscosity, and crystal structure of avocado seed starch. Furthermore, two different extraction solvents (solvent A: Tris, NaCl, and NaHSO_3_; solvent B: sodium bisulfite solution) were applied to extract starch from avocado seeds and it was found that the type of solvent did not influence the functional and rheological properties of avocado seed starches [6]. Additionally, avocado seed starch was modified in an attempt to improve its properties: the crystalline form of avocado seed starch was converted from type A to type C after acetylation and acetylated starch had a lower breakdown value and higher oil absorption when compared to native starch [7]. Lacerda et al. [8] also revealed that oxidizing avocado seed starch with standard sodium hypochlorite reduced gelatinization enthalpy, average roughness, and the degree of relative crystallinity. However, to the best of our knowledge, there is little information on the physicochemical properties of avocado seed starches from different cultivars and no studies on their in vitro digestibilities.

The aim of this study was to explore if there are great differences in seed starches from different avocado cultivars regarding their physicochemical and in vitro digestion properties. Starches were firstly extracted from eight avocado cultivars and their amylose content, morphology, particle size, crystallinity, swelling power, water solubility, thermal and pasting properties, and in vitro digestibility were investigated.

## 2. Materials and Methods

### 2.1. Materials

The seeds of eight cultivars of avocado (*P. americana* Mill.) were provided by Haikou Experimental Station, Chinese Academy of Tropical Agricultural Sciences (Haikou, China). The eight cultivars were cultivated in the same environment at the Germplasm Nursery of Haikou Experimental Station at Danzhou, China, and were named as *Re Xuan 1*, *Re Xuan 2*, *Re You 1*, *Re You 2*, *Pinkerton*, *Reed*, *Zutano*, and *Hass* and numbered as 1, 2, 3, 4, 5, 6, 7, and 8, respectively. All chemicals used were ACS certified.

### 2.2. Avocado Seed Starch Isolation

Starch was extracted from the avocado seeds using a previously described method [6] with some modifications. Avocado seeds were weighed, dehulled, cut into small pieces, and quickly immersed in sodium bisulfite solution (0.5%, *w*/*w*) for about 20 min. Then, the pieces were transferred to a beater and broken into slurry after adding approximately two times the raw seeds weight of distilled water. Then, a dual-enzyme solution (pectinase/cellulase = 1:2, *w*/*w*) was added to allow hydrolysis for 1 h under pH 5.0 and 45 °C. The total amount of enzyme added was 0.22% of the weight of avocado seed. Next, the seed slurry was filtered through a 120-mesh filter cloth, and the filtrate was centrifuged at 3000 rpm for 15 min. The acquired sediment was soaked in sodium hydroxide solution (0.3%, *w*/*w*) for 10 min with magnetic stirring. This slurry was centrifuged again, and the precipitate was washed repeatedly with excessive distilled water until the pH was neutral before it was dried and stored in a desiccator for further use.

### 2.3. Apparent Amylose Content

The detectable amylose contents of avocado seed starches were determined by iodine colorimetry [9] with minor modifications. Briefly, 1 mL of 95% ethanol was used to disperse 100 mg of starch, followed by adding 9 mL of 1 M NaOH, which was then stirred at 100 °C for 10 min and made up to 100 mL with distilled water. Next, 5 mL of the acquired solution was combined with 0.5 mL of 1 M acetic acid solution and 0.5 mL of 0.2% iodine solution. The absorbance of the amylose–iodine complex at the maximum absorption wavelength (720 nm) was then measured. Amylose and amylopectin served as standards, and 0.09 M NaOH solution was used as a control.

### 2.4. Morphological Structure

#### 2.4.1. Light Microscopy

Avocado seed starch suspensions (1%, *w*/*w*) were prepared with 50% glycerol solution (*v*/*v*). One drop of the starch suspension was placed on a glass slide, covered with a coverslip, and observed on a light microscope (Olympus BX-51, Tokyo, Japan) equipped with a cross polarizer and a real-time viewing system (Q-capture Pro, Q-Imaging, Surrey, BC, Canada). Microscopic graphs were taken at 100× magnification under normal light and polarized light as well.

#### 2.4.2. Scanning Electron Microscopy (SEM)

The micromorphological surface structure of avocado seed starches was studied using a scanning electron microscope (model EVO 18, Zeiss, Oberkochen, Germany) with an accelerating voltage of 10.00 kV. Starch samples were scattered on an aluminum stubby double-sided adhesive tape, then coated with a thin layer of gold under vacuum with a gold sputter coater for 3 min before being microscopically examined.

### 2.5. X-ray Diffraction (XRD)

The avocado seed starches were analyzed by XRD using a D/Max-2200 X-ray diffractometer (Rigaku Denki Co. Ltd., Tokyo, Japan). Cu K_α_ radiation was used to power the analyzer at 40 kV and 40 mA. The diffractograms were scanned at a rate of 5°/min from 4° to 40° (2θ). The moisture content of the samples was equilibrated in a dryer for 24 h with a relative humidity of 15%. Relative crystallinity was calculated based on the ratio of the crystalline peak to the total diffractogram area [10].

### 2.6. Fourier Transform Infrared Spectroscopy (FT-IR)

All Fourier transform infrared spectroscopic analyses of the avocado seed starches were performed on a Vector 33 FT-IR spectrophotometer (Bruker Company, Ettlingen, Germany) using an attenuated total reflection method. Samples were mixed with KBr in a ratio of 1:100 (starch/KBr) and then compressed to a sheet. Each spectrum was recorded from 4000 to 200 cm^−1^ with a resolution of 4 cm^−1^ and a total of 64 scans.

### 2.7. Swelling Power and Water Solubility

The swelling power and the water solubility of the avocado seed starches were measured using a previously described method with slight changes [11]. Sample suspensions (2% in distilled water, *w*/*w*) were prepared, heated at 55, 65, 75, 85, and 95 °C for 30 min with continuous shaking, cooled to room temperature, and centrifuged at 3000 rpm for 15 min. The wet weights of the precipitates were measured. The supernatants were collected and oven-dried at 105 °C. Water solubility (%) was determined as the ratio of the weight of the dried supernatant to that of the dry starch. Swelling power (g/g) was determined as the weight ratio of the precipitate to the dry starch.

### 2.8. Differential Scanning Calorimetry (DSC)

The thermal property of the avocado seed starches was determined using a differential scanning calorimeter with Pyris thermal analysis software (DSC 8000, Perkin Elmer, Waltham, MA, USA). About 3 mg of starch and 7 μL of distilled water were added into a standard DSC stainless high-pressure pan that was sealed and equilibrated at room temperature for 12 h before analysis. The pan was then heated from 30 to 120 °C at a rate of 10 °C/min with a nitrogen access speed of 20 mL/s. An empty pan was employed as a reference. The enthalpy change (Δ*H*), onset (*T_o_*), peak (*T_p_*), and conclusion (*T_c_*) temperatures were calculated.

### 2.9. Pasting Properties

The viscosity profile of the avocado seed starches was determined using a Micro Visco-Amylo-Graph (Brabender, Duisburg, Germany). Starch was prepared at a concentration of 6.0% (dry starch basis) with distilled water. The torque measured was 700 cmg. The slurry was heated at a rate of 7.5 °C/min from 30 to 95 °C, held at 95 °C for 5 min, cooled to 50 °C at a rate of 7.5 °C/min, and then held at 50 °C for another 5 min.

### 2.10. In Vitro Digestibility

The digestibility of avocado seed starches was investigated using a slightly modified method of Englyst et al. [12]. The enzyme solution was prepared by mixing pancreatin (1.5225 g, 8 USP) in sodium acetate buffer (7.5 mL, 0.1 M, pH 5.0) with magnetic stirring for 30 min. The mixture was centrifuged for 10 min at 3500 rpm. The supernatant was transferred into a beaker and mixed with 0.75 mL of amyloglucosidase (300 U/mL).

Starch (300 mg) was dispersed with 15 mL sodium acetate buffer (0.2 M, pH 5.2) and 0.75 mL of the enzyme solution as prepared above was added. Enzymatic digestion was performed in a water bath maintained at 37 °C with stirring (170 rpm), and an aliquot (0.1 mL) was collected at 20 min and 120 min after enzymolysis started. The aliquot was immediately mixed with 20 mL of 90% ethanol to deactivate the enzymes and centrifuged afterwards. The glucose content in the supernatant was determined after centrifugation (4500 rpm, 10 min) using the glucose oxidase/peroxidase assay kit. The hydrolyzed starch percentage was calculated by multiplying the glucose content with a factor of 0.9, which is the molar mass conversion from glucose to anhydroglucose (the starch monomer unit). The rapidly digested starch (RDS), slowly digested starch (SDS), and resistant starch (RS) contents were obtained using the formulas below:

(1) $$\mathrm{RDS}\;\left(\%\right)=\left[0.9\times \left(G20-\mathrm{FG}\right)\right]/\mathrm{TS}\times 100\%$$

(2) $$\mathrm{SDS}\;\left(\%\right)=\left[0.9\times \left(G120-\mathrm{FG}\right)\right]/\mathrm{TS}\times 100\%$$

(3) $$\mathrm{RS}\;\left(\%\right)=1-\mathrm{RDS}\;\left(\%\right)-\mathrm{SDS}\;\left(\%\right)$$

where G20 and G120 indicate the amount of glucose released at 20 min and at 120 min, respectively; FG represents the free glucose content; and TS is the total starch content.

### 2.11. Statistical Analysis

All analytical determinations were performed in triplicate. SPSS 17.0 (SPSS Inc., Chicago, IL, USA) was used for statistical analysis. Mean values and standard deviations were analyzed. Duncan’s multiple range test was used to examine the difference in means at a confidence level of 95% (*p* < 0.05). Figures were created with the Origin Program 8.0 (Origin Lab Company, Northampton, MA, USA). Data were reported as the mean ± standard deviation.

## 3. Results and Discussion

### 3.1. Detectable Amylose Content

**Figure 1:**
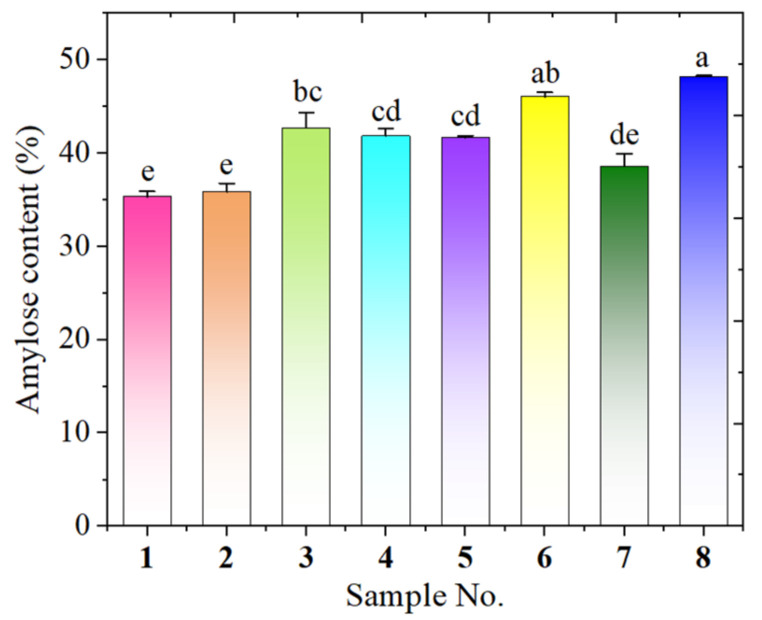
Amylose content of starches from *Re Xuan 1* (**1**), *Re Xuan 2* (**2**), *Re You 1* (**3**), *Re You 2* (**4**), *Pinkerton* (**5**), *Reed* (**6**), *Zutano* (**7**), and *Hass* (**8**). Values with different letters have significant difference (*p* < 0.05).

Starch granules are made up of amylose and amylopectin. The amount of amylose in the starch has a significant impact on its physical and chemical properties, such as rheology, retrogradation, and gelatinization [13]. Therefore, studying the amylose content of starch is crucial for understanding the properties of avocado seed starches. The starch extraction yield ranged from 12.6% to 26.3% and the amylose contents of the eight avocado seed starches were determined and ranged from 35.34 to 48.19% (Figure 1), higher than the data reported for native corn starch (20–25%) [14]. Among them, *Hass* seed starch showed the highest amylose content, while *Re Xuan 1* seed had the lowest amylose content. The observation that amylose content varied with avocado cultivar was in accordance with the data reported by Walter et al. [15], who also discovered that the amylose content of sweet potato starch changed with different sweet potato cultivars. Li et al. [16] also found that the amylose content of starch from different varieties of mung bean varied significantly.

### 3.2. Morphology Observation

**Figure 2:**
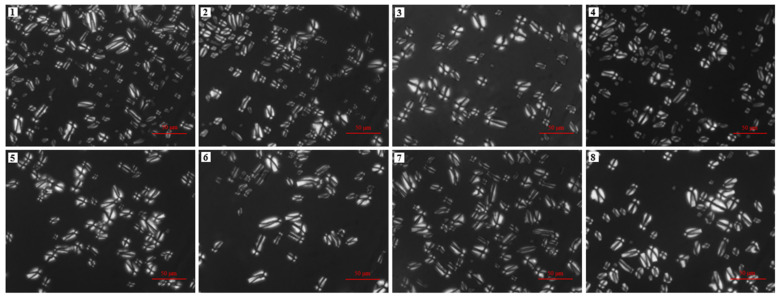
Polarized light microscopic images of starches from *Re Xuan 1* (**1**), *Re Xuan 2* (**2**), *Re You 1* (**3**), *Re You 2* (**4**), *Pinkerton* (**5**), *Reed* (**6**), *Zutano* (**7**), and *Hass* (**8**). Scale bar: 50 μm.

The starch particles exhibit birefringence under a polarizing microscope due to the presence of two different structures (crystal structure and amorphous structures), which are stacked alternately within the starch granules and thus form growth rings. In the present study, a Maltese cross appeared when the eight avocado seed starches were examined under polarized light, with the points of these crosses located at the center or end of the starch particles (Figure 2). This is because of the differences in density and refractive index between the crystalline and amorphous regions when the polarized light passes through the starch particles. No obvious difference was found among these starches.

**Figure 3:**
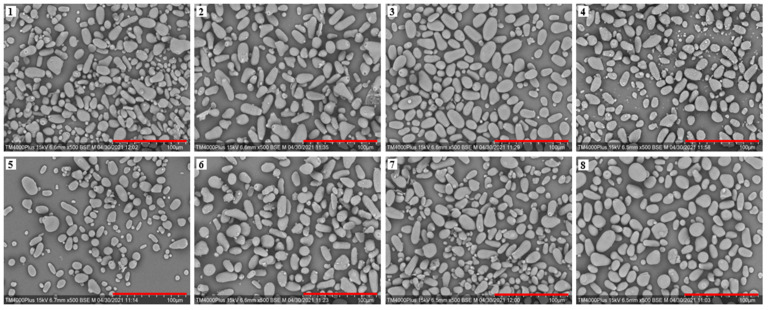
Scanning electron microphotograph (× 500) of starches from *Re Xuan 1* (**1**), *Re Xuan 2* (**2**), *Re You 1* (**3**), *Re You 2* (**4**), *Pinkerton* (**5**), *Reed* (**6**), *Zutano* (**7**), and *Hass* (**8**). Scale bar: 100 μm.

The SEM images of the avocado seed starches are shown in Figure 3. Avocado seed starch granules displayed sizes ranging from 5 to 30 μm. They were found to be smooth, oval, and strip-shaped, which was consistent with the findings of Kahn [5]. The starch granules from the *Re Xuan 1* seed were irregular and not uniform in size, and the majority of them were stripped. Starch from *Re Xuan 2* showed generally larger granules when compared to that from *Re Xuan 1*. The majority of the starch granules from *Re You 1* were oval, and the granule sizes were relatively uniform. Interestingly, the granules of *Pinkerton* starch were the smallest among the samples, with granule sizes ranging from 5 to 20 μm. The shape and size of starch granules are primarily determined by biological factors. It was suggested earlier that the biochemistry of the chloroplast or amyloplast as well as plant physiology determine the morphology of starch granules [17], which could result in the differences for the eight cultivars investigated here. Therefore, the morphology and size of the eight avocado seed starch granules differed.

### 3.3. Crystalline Profile

**Figure 4:**
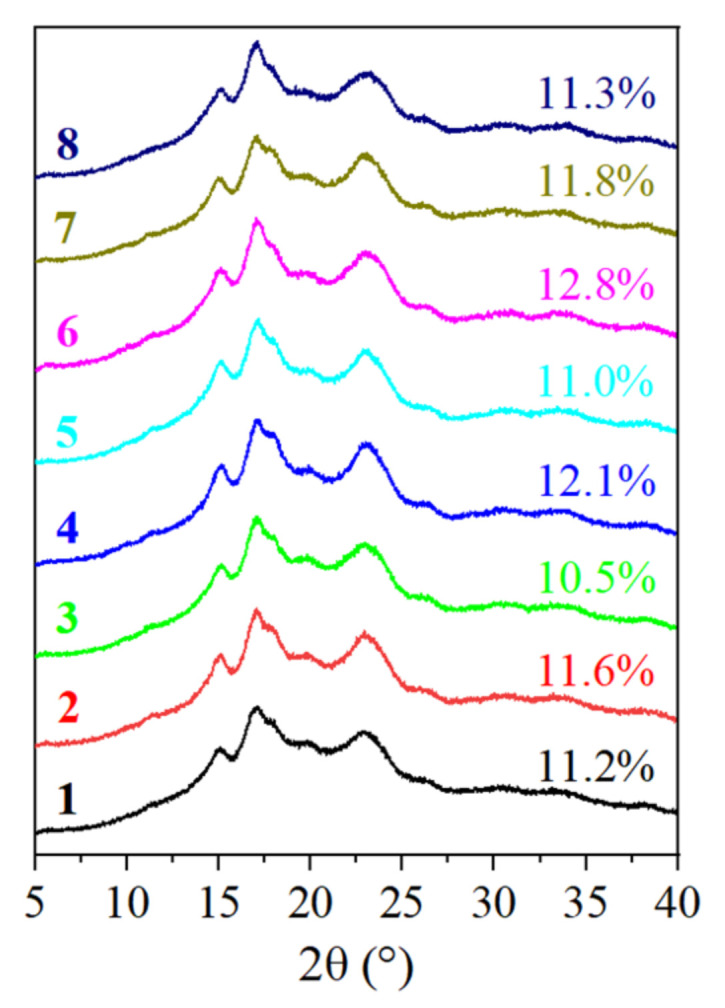
The XRD images of starches from *Re Xuan 1* (**1**), *Re Xuan 2* (**2**), *Re You 1* (**3**), *Re You 2* (**4**), *Pinkerton* (**5**), *Reed* (**6**), *Zutano* (**7**), and *Hass* (**8**).

The crystalline structure in the starch granules is caused by the ordered arrangement of the double helix formed by the branches of amylopectin. The crystalline structure and relative crystallinity of the eight starches were determined by XRD. Their X-ray diffractograms are displayed in Figure 4. Their diffraction curves were similar, demonstrating that the eight avocado seeds had the same type of starch. As illustrated, the avocado seed starches exhibited a typical A-type crystalline pattern with peaks at 15.3°, 17.1°, a shoulder peak to the right of 17.1°, and 23° (2θ). The relative degree of crystallinity of the avocado seed starches ranged between 10.5 and 12.8%. The findings corroborated the results of Lacerda et al. [8], who observed the same type of crystalline structure and calculated the relative crystallinity to be 13.03 ± 0.91% in avocado starch extracted from the seeds of mature fruits obtained from a local market in Curitiba, PR, Brazil. The results demonstrated that the cultivars had little effect on the crystalline structure of the avocado seed starch. A homothetic result was reported by Chung et al. [18], who found no significant difference in the crystalline structure of starch from common bean varieties grown in Canada.

### 3.4. Fourier Transformed Infrared Spectra

**Figure 5:**
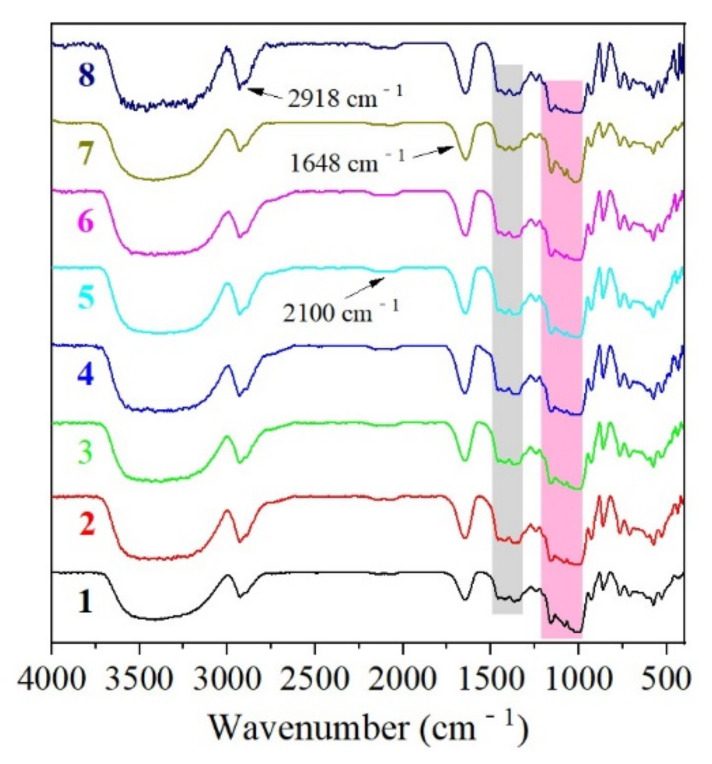
The FT-IR spectra of starches from *Re Xuan 1* (**1**), *Re Xuan 2* (**2**), *Re You 1* (**3**), *Re You 2* (**4**), *Pinkerton* (**5**), *Reed* (**6**), *Zutano* (**7**), and *Hass* (**8**).

All the avocado seed starches had similar FT-IR profiles and no conspicuous difference was observed (Figure 5). They showed three main regions (3600–2900, 1700–1000, 1000–400 cm^−1^) in the spectra. The O-H bond stretching vibration in glucose units was responsible for the extremely broad absorption band from 3600 cm^−1^ to 3000 cm^−1^ [19]. The peak at 2918 cm^−1^ corresponds to an asymmetric C-H stretching vibration [20]. The broad and flat peak at 2100 cm^−1^ was assigned to the remnant free water molecules, whereas the peak at 1648 cm^−1^ could originate from the strongly bound water molecules in the starch, which was suggested to play some structural role [21]. The absorption region from 1340 cm^−1^ to 1500 cm^−1^ (grey shaded area) was related to the C-H bending vibration of CH_2_ and O-H bending of primary or secondary alcohols [19]. Moreover, the absorption peaks in the region from 1200 cm^−1^ to 1000 cm^−1^ (pink shaded area) could be ascribed to the stretching of C-O, C-C, and C-O-H, and C-O-H bending as well from glucose residues [20]. For example, 1048 cm^−1^, 1022 cm^−1^, and 1164 cm^−1^ have been assigned to the crystalline region, amorphous region, and vibration of the glycosidic linkage (C-O-C), respectively [21]. In general, no conspicuous difference was spotted on the spectra of the eight starches, which meant the chemical linkages in them were the same and no dominant linkage was found among the eight starches.

### 3.5. Swelling Power and Water Solubility

The swelling power and the water solubility of starch are proportional to the magnitude of the interaction force between starch molecules and water molecules. The basis of dissolution is the swelling of starch granules and the degree of swelling can reflect the bonding degree of the internal bonds of the particles. The relatively loose amorphous region was suggested to swell first, followed by the amorphous region close to the crystalline region, and finally the crystalline region [22].

**Figure 6:**
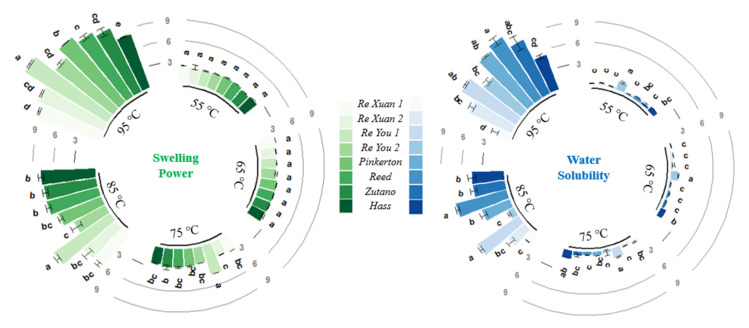
Swelling power and water solubility of starches from *Re Xuan 1* (**1**), *Re Xuan 2* (**2**), *Re You 1* (**3**), *Re You 2* (**4**), *Pinkerton* (**5**), *Reed* (**6**), *Zutano* (**7**), and *Hass* (**8**). Values with different letters in the same cluster have significant difference (*p* < 0.05).

Figure 6 depicts the swelling power and the water solubility of the avocado seed starches. The swelling power of all the avocado seed starches increased as the temperature went up. When starch suspensions are subjected to high temperatures, the double helices from amylopectin in the granule are disassembled, causing the granules to swell quickly and amylose to preferentially leach out from the swollen granules. Moreover, amylose could reduce granule swelling and keep the swollen starch granules in place [23]. In the present study, the swelling power of these starches changed little when the temperature was lower than 85 °C but soared after reaching 85 °C. This trend was in agreement with water solubility: it changed slowly with the temperature when the temperature was lower than 85 °C, whereas it increased dramatically when reaching 85 °C and thereafter. This could be attributed to starch gelatinization and the destruction of most of the particles. Extensive water could be absorbed into the granules and crystallinity was lost gradually during gelatinization. This led to the swelling and increased the volume of the starch granules as well as the amount of leached amylose. With respect to all the avocado seed starches, the Re You 1 starch had the highest swelling power at 85 °C and 95 °C, and it showed great water solubility accordingly. Amylose exudation in expanded starch granules could influence water solubility [24]. Herein, the latter six samples (**3–8**) displayed obviously higher amylose content than the first two starches (Figure 1) and, accordingly, they exhibited higher water solubility than the first two starches on the whole.

### 3.6. Thermal Properties

Table 1 summarizes the thermal parameters of the avocado seed starches. Among the avocado seed starches, the *Pinkerton* starch exhibited the highest gelatinization temperatures (T_o_ = 82.38 ± 0.83 °C, T_p_ = 87.58 ± 0.72 °C, and T_c_ = 94.45 ± 0.35 °C), whereas the *Re You 1* starch presented the lowest gelatinization temperatures (T_o_ = 75.02 ± 0.25 °C, T_p_ = 78.49 ± 0.47 °C, and T_c_ = 83.67 ± 0.82 °C). Notably, all these temperatures were higher than those of maize starch (T_o_ = 65.6 °C, T_p_ = 70.4 °C, and T_c_ = 75.2 °C) [25] and cassava starch (T_o_ = 64.26 °C, T_p_ = 71.70 °C, and T_c_ = 81.00 °C) [26]. The gelatinization temperatures agreed well with our observations on swelling power and solubility determination. We noticed that the gelatinization temperatures of avocado seed starches reported by Chel-Guerrero et al. [6] were lower (56–74 °C) than those in this study. This could be because the avocado seed starches in this study had higher amylose contents (35.34–48.19%) than those studied by Chel-Guerrero et al. [6], who reported the amylose content to be 15–16%. Starch granules have a typical polycrystalline structure, consisting of both crystalline and amorphous regions. Granules begin amorphization and gelatinization upon absorption of heat from surrounding water to a certain temperature. The compactness of starch granules may affect T_p_ during gelatinization. The endotherm of the crystalline part during the gelatinization process results in a melting peak in the thermogram of starch. Starches with a high amylose content usually have a higher crystallinity and thus denser structure, resulting in higher melting and gelatinization temperatures because more energy is required to initiate the dislocation or breakage of orderly and firmly bound glycosidic bonds [27].

The Δ*H* of the eight avocado seed starches was between 10.12 and 15.26 J/g. Starch gelatinization enthalpies determined by DSC may be related to many starch granule characteristics such as crystallinity [28]. A high degree of crystallinity provides structural stability and makes the granules more resistant to gelatinization [29]. The *Reed* starch showed the highest relative crystallinity among the avocado seed starches. As such, the Δ*H* of the *Reed* starch was in the greatest group among the avocado seed starches.

### 3.7. Pasting Properties

The pasting characteristics of the starches isolated from *Re Xuan 1*, *Re Xuan 2*, *Re You 1*, *Re You 2*, *Pinkerton*, *Reed*, *Zutano*, and *Hass* are displayed in Table 2. The gelatinization initiation temperature is the temperature at which starch begins to gelatinize and starch granules begin to swell. The peak pasting temperature is reached when the starch granule structure stops expanding, causing the starch granule to become deformed or broken. Of the samples tested in this study, although the *Hass* starch had the highest gelatinization initiation temperature and the *Re You 1* starch had the lowest among the eight avocado seed starches, the values were found to be statistically insignificant. The pasting temperature acquired here was also consistent with that from DSC analysis and solubility experiment. Peak viscosity is an important characteristic of starch because it could be used to differentiate starch. The *Re You 1* starch was found to have the highest peak viscosity. The final viscosity of these starches ranged from 290 ± 4.5 to 528 ± 2.7 cP, which corresponded to *Re Xuan 2* and *Re You 1*, respectively. The final viscosity represents the stability of the swollen granule structure, and high values may be because linear amylose restricted the swelling of starch granules. The differences in pasting characteristics among all the avocado seed starches could be ascribed to different rheological and morphological properties of the starch granules [30]. The avocado seed starches had breakdown values of about 0–5 cP, indicating that their viscosities barely decreased during processing stages. They also showed low setback values (107–216 cP), suggesting that they were stable during heating and cooling processes.

### 3.8. In Vitro Digestibility

RDS, SDS, and RS contents of starches from *Re Xuan 1*, *Re Xuan 2*, *Re You 1*, *Re You 2*, *Pinkerton*, *Reed*, *Zutano*, and *Hass* are shown in Table 3. The RDS contents ranged from 6.20 ± 1.15% to 21.69 ± 0.08%, which were significantly lower than the RDS contents of corn starch (24.4%), wheat starch (40.1%), and rice starch (32.4%) [31]. On the contrary, the RS fractions ranged from 63.82 ± 1.69% to 77.83 ± 1.10%, which were higher than the RS contents of most uncooked native starches, including waxy corn starch (4.8%), normal corn starch (9.4%), Hylon Ⅴ (29.0%) and Hylon Ⅶ (30.7%) [32], pea starch (10.0%), lentil starch (9.1%) [33], and wheat starch (7.9%) [34]. It has been demonstrated that several factors, including starch source, amylose content, particle size, degree of crystallinity [35], and amylopectin fine structure [36], could influence raw starch digestibility. Following the above analysis, the amylose contents of starch from *Re You 1*, *Re You 2*, *Pinkerton*, *Reed*, and *Hass* were higher than those from *Re Xuan 1*, *Re Xuan 2*, and *Zutano* in the present study, which showed consistency with the RS content acquired here. At the same time, Hass had the highest amylose content, and it presented the highest RS content here. In agreement with our finding, Vatanasuchart et al. [37] reported a linear positive relationship between apparent amylose content and RS content (R^2^ = 0.76). Large starch granule size is associated with lower digestibility as it has a smaller specific surface area and thus provides fewer binding sites for digestive enzymes. The granule size of avocado starch in the present study is relatively larger than that of corn starch in [32], which might be one of the reasons the eight starches have higher RS content than corn starch. Moreover, the compactness of starch granules can play a significant role in digestibility. Corn starch, for example, is known for its loose structure because of the presence of cavities inside the granules, resulting in an “inside out” hydrolytic mode and high digestibility. Herein, despite crater-like pits existing at the end of some granules (Figure 3), the surface of the avocado starch granules is even and smooth and resembles that of potato starch, which has a lower degree of enzymatic hydrolysis when uncooked in comparison to high amylose corn starch [38]. Therefore, the avocado starch may have a compact structure that contributes to enzymatic resistance. However, in light of the lower crystallinity and comparative amylose content to that of the pea, lentil, and wheat starch [33,34], perhaps we should delve more into the fine structure of amylopectin in the avocado starches. Short linear chains can serve as branching points within the amylopectin that may juxtapose with each other and become resistant to α-amylase action [36,38]. With the high RS content observed here, it is possible that the starches have such structures. Systematic work is required to investigate the ordered structure or supramolecular arrangements of starch chains and verify these assumptions. Nonetheless, this falls out of the scope of the present study.

Apart from the starch structure, the method per se could also induce differences in RS content, which can be reflected by comparing the results of Aparicio-Saguilán et al. [39], Tribess et al. [40], and Vatanasuchart et al. [37] concerning the RS content in green banana starch. In this study, 0.75 mL enzyme solution was used for the in vitro digestibility analysis, lower than that used in [33,34]. This could bring relatively higher RS content. It is worthwhile to mention that the “RDS, SDS, and RS” system cannot accurately estimate the amount of starch that reaches the terminal ileum since it fails to give full consideration to gastric residence time, small intestine passage rate, and many other factors, as pointed out by Dhital et al. [38]. However, we assume a certain credibility of the results obtained by the current evaluation system because it can at least provide preliminary hints on the potential of introducing RS into our digestive tract. Admittedly, more efforts need to be made in the future to more accurately assess the potential of using avocado seed starch as a source of RS.

## 4. Conclusions

The physiochemical and functional properties of starches extracted from the seeds of eight cultivars of avocado were systematically investigated in this work. The XRD and FT-IR analyses revealed that cultivar had little influence on the crystalline structure and chemical linkage of the avocado seed starch. However, many other properties varied with different varieties, including amylose content, granule size, swelling power, water solubility, and pasting as well as thermal properties. Interestingly, the RS fractions of these avocado seed starches ranged from 63 to 78%, which was much higher than that of most uncooked native starches. This study provides valuable information on projecting the possibility of making use of avocado seeds (a food waste) for the commercial production of RS and latent related food products.

## Figures and Tables

**Table 1:** Differential scanning calorimetry results for gelatinization of starches isolated from Re *Xuan 1* (**1**), *Re Xuan 2* (**2**), *Re You 1* (**3**), *Re You 2* (**4**), *Pinkerton* (**5**), *Reed* (**6**), *Zutano* (**7**), *Hass* (**8**).

Sample No.	T_o_ (°C)	T_p_ (°C)	T_c_ (°C)	Δ*H* (J/g)
1	80.89 ± 0.84 ^a^	85.33 ± 0.65 ^b^	92.09 ± 0.72 ^c^	11.07 ± 0.63 ^de^
2	81.89 ± 0.51 ^a^	86.39 ± 0.8 ^ab^	93.10 ± 0.64 ^bc^	12.51 ± 0.95 ^bcde^
3	75.02 ± 0.25 ^b^	78.49 ± 0.47 ^c^	83.67 ± 0.82 ^d^	10.12 ± 0.59 ^e^
4	81.44 ± 0.61 ^a^	85.87 ± 0.58 ^ab^	93.25 ± 0.84 ^bc^	11.47 ± 0.41 ^cde^
5	82.38 ± 0.83 ^a^	87.58 ± 0.72 ^a^	94.45 ± 0.35 ^ab^	15.26 ± 0.69 ^a^
6	81.54 ± 0.56 ^a^	85.99 ± 0.65 ^ab^	95.71 ± 0.13 ^a^	13.93 ± 0.76 ^abc^
7	80.41 ± 0.79 ^a^	85.05 ± 0.15 ^b^	91.19 ± 0.39 ^c^	15.03 ± 0.90 ^ab^
8	80.72 ± 0.40 ^a^	85.32 ± 0.51 ^b^	91.61 ± 0.62 ^c^	13.59 ± 0.95 ^abcd^

Values in the same column with different superscript letters are significantly different (*p* < 0.05). T_o_, T_p_, and T_c_ indicate the onset, peak, and conclusion temperature of gelatinization, respectively. Δ*H* indicates the enthalpy of gelatinization.

**Table 2:** Pasting characteristics of starches isolated from *Re Xuan 1* (**1**), *Re Xuan 2* (**2**), *Re You 1* (**3**), *Re You 2* (**4**), *Pinkerton* (**5**), *Reed* (**6**), *Zutano* (**7**), and *Hass* (**8**).

Sample No.	T_p_ (°C)	*η_pk_* (cP)	*H_f_* (cP)	*η_bd_* (cP)	*η_sb_* (cP)
1	84.2 ± 1.2 ^a^	208 ± 1 ^c^	401 ± 1 ^b^	0	216 ± 6 ^a^
2	84.3 ± 1.3 ^a^	185 ± 2 ^d^	290 ± 5 ^f^	1 ± 1 ^b^	107 ± 3 ^f^
3	80.2 ± 3.2 ^a^	350 ± 4 ^a^	528 ± 3 ^a^	5 ± 1 ^a^	170 ± 4 ^c^
4	85.1 ± 2.0 ^a^	234 ± 4 ^b^	376 ± 1 ^c^	0	134 ± 4 ^d^
5	82.6 ± 1.6 ^a^	235 ± 14 ^b^	384 ± 2 ^c^	0	131 ± 6 ^d^
6	83.6 ± 0.8 ^a^	232 ± 2 ^b^	350 ± 3 ^d^	1 ± 1 ^b^	109 ± 7 ^f^
7	84.8 ± 2.9 ^a^	209 ± 6 ^c^	324 ± 5 ^e^	0	117 ± 4 ^e^
8	87.1 ± 4.1 ^a^	131 ± 4 ^e^	332 ± 6 ^e^	0	184 ± 3 ^b^

Values followed by different superscript letters within a column differ significantly (*p* < 0.05). T_p_, pasting temperature; *η*_pk_, peak viscosity; *η*_f_, final viscosity; *η*_bd_, breakdown viscosity; and *η*_sb_, setback viscosity. The unit of pasting properties of avocado starch is expressed as cP, where cP is the unit of viscosity from the Modular Compact Rheometer.

**Table 3:** In vitro digestibility of starches isolated from *Re Xuan 1* (**1**), *Re Xuan 2* (**2**), *Re You 1* (**3**), *Re You 2* (**4**), *Pinkerton* (**5**), *Reed* (**6**), *Zutano* (**7**), and *Hass* (**8**).

Sample No.	RDS (%)	SDS (%)	RS (%)
1	8.71 ± 0.81 ^e^	27.47 ± 0.88 ^a^	63.82 ± 1.69 ^e^
2	6.20 ± 1.15 ^f^	28.01 ± 2.59 ^a^	65.79 ± 1.44 ^e^
3	13.47 ± 1.01 ^c^	13.07 ± 1.69 ^c^	73.46 ± 0.67 ^bc^
4	21.69 ± 0.08 ^a^	6.52 ± 0.42 ^e^	71.79 ± 0.34b ^cd^
5	19.11 ± 0.93 ^b^	9.52 ± 1.07 ^de^	71.37 ± 0.14 ^cd^
6	18.87 ± 0.47 ^b^	6.85 ± 0.62 ^e^	74.28 ± 0.14 ^b^
7	10.10 ± 0.65 ^de^	20.26 ± 0.76 ^b^	69.64 ± 1.41 ^d^
8	10.70 ± 0.20 ^d^	11.47 ± 1.29 ^cd^	77.83 ± 1.10 ^a^

Values with different superscript letters in the same column have significant difference (*p* < 0.05).

## Data Availability

All data are contained within the article.
